# Compression of the posterior interosseous nerve secondary to a synovial cyst: Case report and review of the literature

**DOI:** 10.1016/j.ijscr.2023.109115

**Published:** 2023-12-06

**Authors:** Ayoub Boushabi, Hicham Aitbenali, Mohammed Shimi

**Affiliations:** Department of Orthopedics and Trauma-surgery, Mohammed VI University Hospital, Faculty of Medicine and Pharmacy, Tangier, Morocco

**Keywords:** Posterior interosseous nerve, Cyst, Neurolysis

## Abstract

**Introduction and importance:**

Posterior interosseous nerve syndrome secondary to compression by a synovial cyst at the elbow is a rare and often unrecognized pathology. Early management relies on complete neurolysis to achieve satisfactory functional recovery. Increasing awareness among the orthopedics will help in the early diagnosis of the disease and in the initiation of early and proper treatment.

**Case presentation:**

In this article, we report the case of a 32-year-old patient with posterior interosseous nerve syndrome secondary to compression by a synovial cyst of the elbow. Surgical management combined with post-operative rehabilitation resulted in indolence with good functional recovery.

**Clinical discussion:**

Posterior interosseous nerve syndrome secondary to compression by a synovial cyst at the elbow is a rare entity. Anatomically, the deep branch of the radial nerve or posterior interosseous nerve passes through the Fröhse's arch or arch of the supinator muscle at the elbow, then travels between the two heads of this muscle. Several anatomical structures may compress the NIOP. Clinically, it presents as paralysis or paresis of the extensor muscles of the fingers and the abductor muscle of the thumb. Limitation of the ulnar extensor carpi may be responsible for radial deviation of the carpus in some cases. MRI is the radiological examination of choice. Electromyography plays a contributory role in diagnosis prior to surgical exploration. Surgical excision is the treatment of choice. It may be combined with radial neurolysis for better recovery. Progression after surgical treatment is generally favourable.

**Conclusion:**

Ignorance of posterior interosseous nerve palsy syndrome frequently leads to misdiagnosis. Early management relies on complete neurolysis to achieve satisfactory functional recovery.

## Highlights

• Posterior interosseous nerve syndrome is a very rare condition.
• Several anatomical structures are likely to compress this nerve along its course, and this compression may also be due to a lipoma or an expansive process of articular origin (synovitis, synovial cyst).
• Clinically, it can take two forms: one sensitive and the other purely motor, or posterior interosseous nerve syndrome caused by compression at Fröhse's arch [1].
• Ignorance of posterior interosseous nerve palsy syndrome frequently leads to misdiagnosis.
• Early management relies on complete neurolysis to achieve satisfactory functional recovery.

## 1 Introduction

Posterior interosseous nerve syndrome is a very rare condition. Several anatomical structures are likely to compress this nerve along its course, and this compression may also be due to a lipoma or an expansive process of articular origin (synovitis, synovial cyst).

Clinically, it can take two forms: one sensitive and the other purely motor, or posterior interosseous nerve syndrome caused by compression at Fröhse's arch [1]. In this article, we present a rare case of a patient with posterior interosseous nerve syndrome secondary to a surgically treated synovial cyst of the right elbow. The course was marked by improvement in symptomatology and good functional recovery.

## 2 Observation

**Fig. 1:**
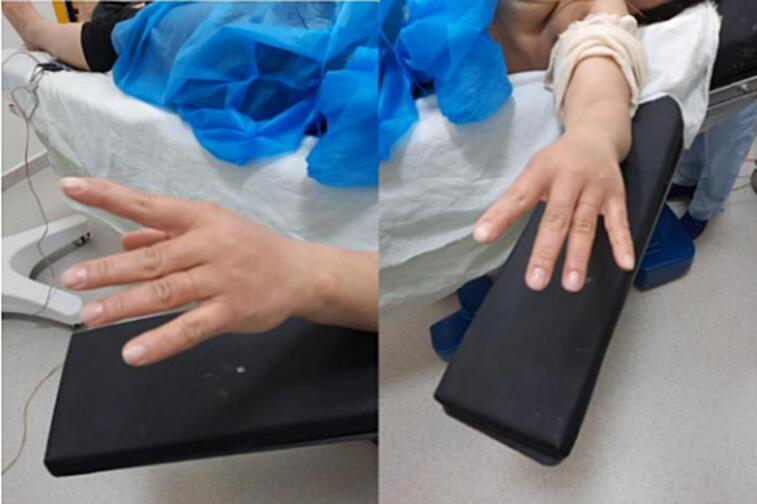
Clinical examination showing limited finger extension and slight radial deviation of the wrist in active extension.

**Fig. 2:**
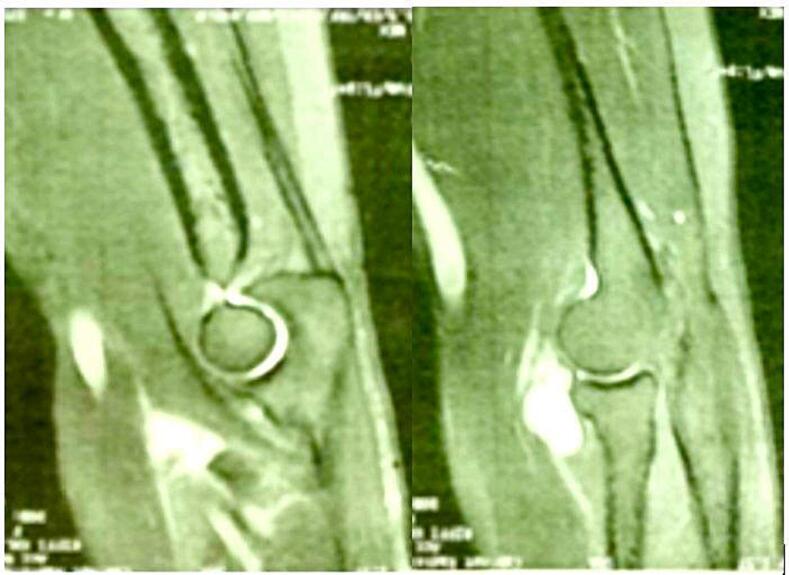
MRI of the elbow showing the synovial cyst compressing the deep branch of the radial nerve at elbow level.

**Fig. 3:**
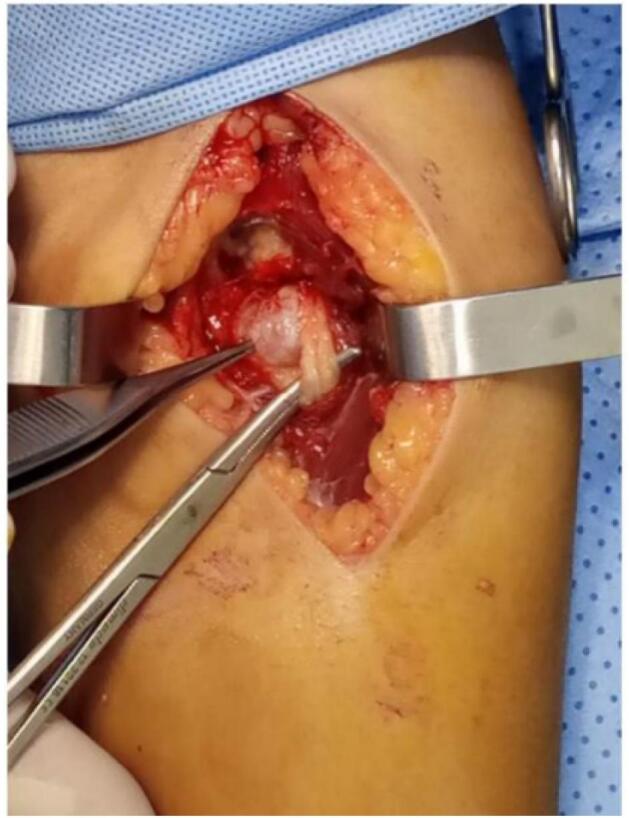
Intraoperative appearance of the synovial cyst compressing the deep branch of the radial nerve.

**Fig. 4:**
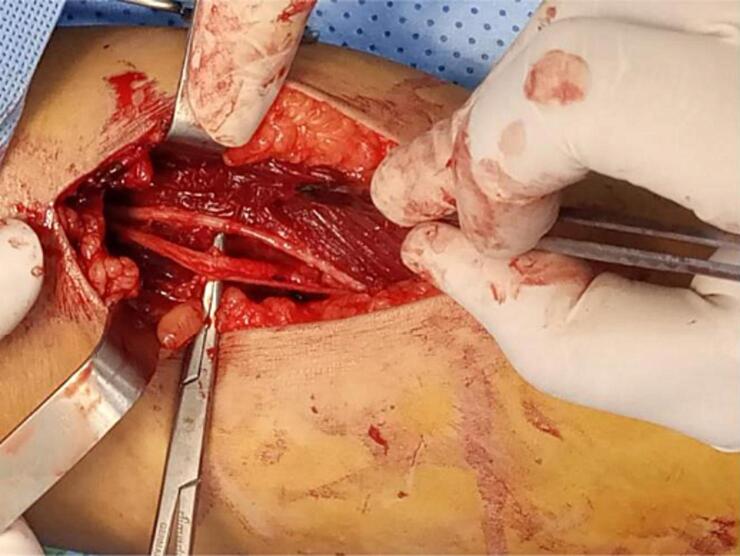
Intraoperative appearance after resection of the synovial cyst and radial nerve neurolysis.

**Fig. 5:**
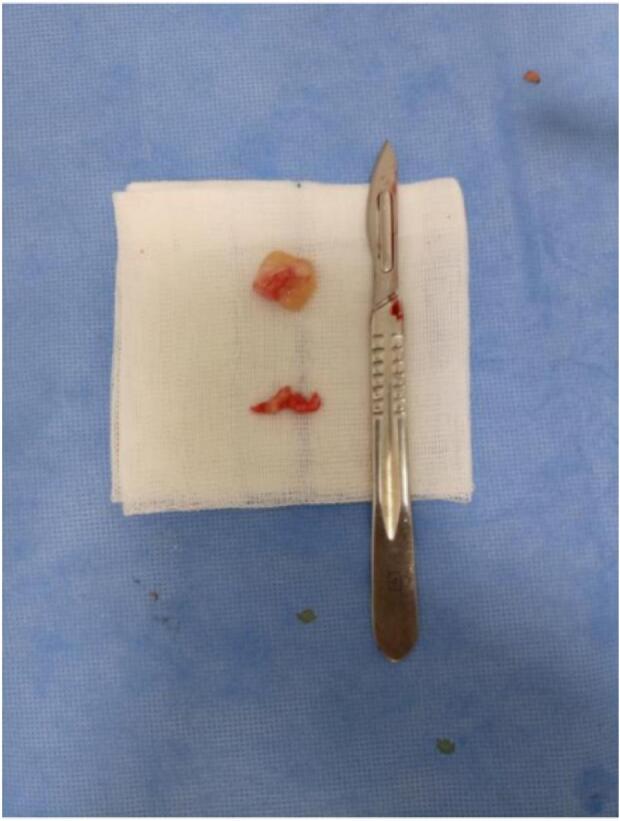
Macroscopic appearance of the synovial cyst.

This is a 32-year-old patient, right-handed, manual worker, with no particular pathological history, who consulted in rheumatology for chronic pain in the left elbow progressively evolving for 6 months, associated with tingling of the lateral face of the forearm and a deficit of extension of the fingers. The clinical examination objectified a limitation of the extension of the fingers (especially the 3rd, 4th and 5th) with a slight radial deviation of the wrist in active extension (Fig. 1). Sensitivity examination revealed paresthesias in the territory of the posterior interosseous nerve. The electromyographic examination showed a conduction block on the posterior interosseous nerve (NIOP) at the level of the elbow. MRI of the elbow revealed a mass with a cystic aspect measuring 2 cm × 1.5 cm in contact with the deep branch of the radial nerve coming from the anterior aspect of the elbow joint (Fig. 2). Using an anterolateral approach, surgical exploration of the course of the deep branch of the right radial nerve opposite the site of compression revealed a synovial cyst compressing the branch (Fig. 3). Complete removal of the cyst was performed, with neurolysis of the radial nerve (Figs. 4, 5). Histological study confirmed the diagnosis of synovial cyst. The recovery has been complete, with the resolution of tingling on the lateral aspect of the forearm and the disappearance of paresthesia in the territory of the posterior interosseous nerve. The patient has regained full finger extension function, especially of the third, fourth, and fifth fingers.

Methods: This work has been reported in line with the SCARE criteria.

## 3 Discussion

Posterior interosseous nerve syndrome secondary to compression by a synovial cyst at the elbow is a rare entity [2]. Anatomically, the deep branch of the radial nerve or posterior interosseous nerve passes through the Fröhse's arch or arch of the supinator muscle at the elbow, then travels between the two heads of this muscle [1]. NIOP syndrome was first described by D.H. Agnew in 1863 [3], and affects males on the dominant side [4].

Several anatomical structures may compress the NIOP [3,5,6]. Most commonly, the proximal edge of the superficial head of the supinator muscle, or Fröhse's arch, is implicated. However, the following have also been implicated: the anteromedial border and deep fascia of the extensor carpi radialis brevis, fibrous formations in the humero-radial joint capsule, transverse branches of the radial recurrent artery and the distal border of the supinator. The NIOP may also be compressed in rare cases by an expansive process of articular origin (synovitis, synovial cyst) or a soft-tissue tumor (lipoma) [7,8]. In 2013, Kohyama et al. reported a case of posterior interosseous nerve compression by pigmented villonodular synovitis of the elbow [9].

Clinically, it presents as paralysis or paresis of the extensor muscles of the fingers and the abductor muscle of the thumb. Elbow and wrist extension is generally preserved. Limitation of the ulnar extensor carpi may be responsible for radial deviation of the carpus in some cases [10].

MRI is the radiological examination of choice. It allows us to study the relationships and local extension of the tumor mass. Electromyography plays a contributory role in diagnosis prior to surgical exploration [11], as it can be used to detect conduction blocks and neurogenic tracings in the affected muscles (extensor digitorum, extensor carpi ulnaris, abductor pollicis longus, extensor pollicis longus and extensor pollicis brevis).

Surgical excision is the treatment of choice. It may be combined with radial neurolysis for better recovery [12,13]. Progression after surgical treatment is generally favourable [14].

## 4 Conclusion

Ignorance of posterior interosseous nerve palsy syndrome frequently leads to misdiagnosis. Early management relies on complete neurolysis to achieve satisfactory functional recovery.

## 5 Methods

This work has been reported in line with the SCARE 2023 criteria.

## Ethical approval

Not applicable.

## Sources of funding

This research did not receive any specific grant from funding agencies in the public, commercial, or not-for-profit sectors.

## CRediT authorship contribution statement

BOUSHABI Ayoub: study concept, Data collection; data analysis; writing review & editing.

AITBENALI Hicham: Contributor, Supervision and data validation.

SHIMI Mohammed: supervision and data validation.

## Guarantor

BOUSHABI Ayoub.

SHIMI Mohammed.

## Registration of research studies

As this manuscript was a case report with no new medical device nor surgical techniques, not prior registration is required.

## Consent

Written informed consent was obtained from the patient for publication and any accompanying images. A copy of the written consent is available for review by the Editor-in-Chief of this journal on request.

## Declaration of competing interest

The authors state that they have no conflicts of interest for this report.
